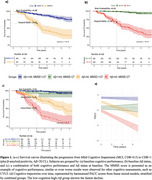# Predicting Mild Cognitive Impairment to Dementia Progression: Optimizing Aβ PET and MMSE Cutoffs

**DOI:** 10.1002/alz70859_104130

**Published:** 2025-12-25

**Authors:** Rosita Shishegar, Pierrick Bourgeat, Vincent Dore, James D. Doecke, Rodrigo Canovas, Simon M. Laws, Tenielle Porter, Azadeh Feizpour, Michael W Weiner, Colin L Masters, Paul Maruff, Hamid R Sohrabi, Jurgen Fripp, Victor L. Villemagne, Christopher C. Rowe

**Affiliations:** ^1^ The Australian e‐Health Research Centre, CSIRO, Melbourne, VIC Australia; ^2^ Department of Electrical and Computer Systems Eng., Monash University, Monash, VIC Australia; ^3^ CSIRO Health and Biosecurity, Australian E‐Health Research Centre, Brisbane, QLD Australia; ^4^ Department of Molecular Imaging, Austin Health, Melbourne, VIC Australia; ^5^ CSIRO, Melbourne, VIC Australia; ^6^ The Australian e‐Health Research Centre, CSIRO, Brisbane, QLD Australia; ^7^ Australian E‐Health Research Centre, CSIRO, Melbourne, VIC Australia; ^8^ Centre for Precision Health, Edith Cowan University, Joondalup, Western Australia Australia; ^9^ Department of Molecular Imaging & Therapy, Austin Health, Melbourne, VIC Australia; ^10^ The Florey Institute of Neuroscience and Mental Health, Parkville, VIC Australia; ^11^ San Francisco Veterans Administration Medical Center (SFVAMC), San Francisco, CA, CA USA; ^12^ The Florey Institute of Neuroscience and Mental Health, The University of Melbourne, Parkville, Melbourne, VIC Australia; ^13^ Cogstate Ltd., Melbourne, VIC Australia; ^14^ Murdoch University, Murdoch, Western Australia Australia; ^15^ University of Pittsburgh School of Medicine, Pittsburgh, PA USA; ^16^ Department of Nuclear Medicine and Centre for PET, Austin Health, Heidelberg, Vic, Heidelberg, VIC Australia; ^17^ Department of Nuclear Medicine and Centre for PET, Austin Health, Heidelberg, Vic, Australia, Heidelberg, VIC Australia; ^18^ Florey Department of Neuroscience and Mental Health, University of Melbourne, Parkville, VIC Australia

## Abstract

**Background:**

This study aims to determine the optimal threshold for Aβ PET to identify individuals with mild cognitive impairment (MCI) who are at high risk of progressing to Alzheimer's disease (AD). Additionally, it assesses whether combining β‐amyloid with Mini‐Mental State Examination (MMSE) performance can enhance risk stratification in MCI which can guide clinical decision‐making regarding early therapeutic interventions.

**Methods:**

We included 686 MCI participants with CDR 0.5 from two cohorts, followed for up to 7 years. Harmonized data from AIBL (N=166) and ADNI (N=520) were analysed using Cox proportional‐hazards models, adjusted for sex, and APOE4 status, with the event of interest being progression to mild dementia due to AD (detected by CDR = 1 and CL>20). Optimal thresholds for MMSE (27) and Aβ (44 CL) were selected to maximize hazard ratios (HR) at 3 years, categorizing participants into low‐risk and high‐risk groups based on cognitive performance and Aβ load. Note that the MMSE score was selected as it is frequently used in clinical practice and in trials.

**Results:**

Both thresholds showed comparable hazard ratios (Figure 1). However, the MCI high‐cognition group had a significantly higher risk of progressing to AD (measured with risk probability (RP)=1‐survival probability; RP=0.08±0.05) than MCI low‐Aβ (RP=0.01±0.01). Combining both cutoffs improved risk stratification: 51 out of 135 MCI low‐cognition, high‐Aβ progressed to AD within 3 years (50% survival probability, HR=2.00), while only 1 of 308 of the MCI high‐cognition low‐Aβ progressed to AD (RP=0.00±0.01). Furthermore, we tested other cognitive assessments, such as CVLT, which provided similar or even statistically worse results in comparison to MMSE. The low‐cognition high‐Aβ group showed the fastest decline, with an annual rate of decline of 0.34 PACC scores, and an effect size of 0.75, compared to the high‐cognition, low‐Aβ group.

**Conclusion:**

While cognitive performance alone is not sufficient for predicting MCI‐to‐AD progression, combining Aβ with MMSE cutoffs can enhance risk stratification, providing greater prognostic information and aiding in the design of clinical trials and therapeutic interventions for prodromal AD. This study also highlights the importance of using CL>44 to identify individuals at high risk of progressing to AD.